# Chain mediation of anxiety and suicidal ideation: a 2021–2025 cross-sectional analysis in Chinese temporomandibular disorders patients

**DOI:** 10.1186/s12888-025-07020-x

**Published:** 2025-07-07

**Authors:** Ruopeng Zhao, Zheng Ye, Hongyu Ming, Jingran Peng, Po-Kam Wo, Haolun Yang, Liming Zhang, Xin Xiong

**Affiliations:** 1 Department of Radiology, West China Hospital, Sichuan University, Chengdu, Sichuan China; 2 State Key Laboratory of Oral Diseases & National Center for Stomatology & National Clinical Research Center for Oral Diseases, West China Hospital of Stomatology, Sichuan University, No. 14 Section 3, Renmin South Road, Chengdu, 610041 Sichuan China; 3 College of Medical Technology, West China Hospital of Sichuan University, Chengdu, Sichuan China; 4 Rehabilitation Medicine Center, Department of Rehabilitation Medicine, West China Hospital, Sichuan University, Chengdu, 610041 Sichuan China

**Keywords:** Temporomandibular disorders, Anxiety, Orofacial pain, Suicide prevention, Quality of life, Suicidal ideation

## Abstract

**Background:**

Chronic musculoskeletal disorders are risk factors for suicidal ideation (SI). As a musculoskeletal condition, temporomandibular disorders (TMDs) are frequently associated with pain, psychological distress, and reduced quality of life, all of which may contribute to SI. However, the study of SI in TMDs is relatively scarce, and its influencing factors and pathways of impact have not been fully explored. This study aimed to investigate the factors affecting SI and the pathways of its influence.

**Methods:**

This cross-sectional study included 934 TMDs patients and collected demographic information, anxiety levels, pain severity, and TMD-related quality of life (TMD-QoL) using questionnaires. Correlation and mediation analyses were conducted to explore their relationships.

**Results:**

Anxiety was associated with SI (OR = 6.74, 95% CI [3.46–13.13], *p* < 0.001), even after adjustment (OR = 6.70, 95%CI [3.42–13.09], *p* < 0.001). And multiple domains of the TMD-QoL, such as psychological discomfort (OR = 3.61, 95%CI [2.19–5.96], *p* < 0.001), social disability (OR = 4.66, 95%CI [2.67–8.13], *p* < 0.001), were linked to SI. The association between anxiety on SI was mediated by pain (0.11, 95%CI [0.07–0.15], *p* < 0.001) and impact on TMD-QoL (0.25, 95%CI [0.18–0.32], *p* < 0.001).

**Conclusions:**

This study underscores the need to incorporate psychological screening into the clinical management of TMDs to identify risk factors for SI. However, this study is limited by its cross-sectional design, which precludes the exploration of causal relationships. Future longitudinal studies are necessary to further validate these pathways.

## Introduction

Temporomandibular disorders (TMDs) are a group of conditions that affect the temporomandibular joint and its associated myofascial structures, causing limited movement and pain in the joint region [1]. The pathogenesis of TMDs is multifactorial, involving the interaction of biological, psychological, and social factors [2]. Epidemiological data indicate that the prevalence of TMDs in the general population ranges from 5.3 to 12.0% [3]. But it is elevated in anxiety groups [4, 5]. Furthermore, patients with comorbid TMDs and anxiety are more likely to experience symptom catastrophizing and reduced pain thresholds compared to those with TMDs alone [6, 7]. Such findings underscore the critical role of anxiety in the etiology and clinical management of TMD.

Psychological problems are risk factors for suicidal ideation (SI), which refers to an individual’s thoughts or plans regarding suicidal behavior that precede suicidal acts [8]. Studies indicate that untreated psychological disorders have been shown to increase the risk of suicide nearly 20-fold [9]. And the prevalence of SI in some chronic pain patients could reach 28.3% [10]. Given that TMD is characterized by the coexistence of psychological disturbances and pain, psychological disorders associated with TMD may not only exacerbate physical symptoms but also serve as a potential trigger for SI.

Current research on SI in patients with TMDs and other chronic pain conditions remains limited, which predominantly uses SI as a measure of disease assessment. Redding et al. [11] examined differences in SI between unexplained and medically explainable pain, reporting a 1.60-fold higher incidence of SI in the former. Similarly, Wang et al. [12] found that medication overuse headache could increase SI risk (OR = 1.75) in a chronic headache cohort. However, these studies did not explore the underlying factors contributing to SI within these disorders. In TMDs, Heo et al. [13] and Park et al. [14] have consistently reported sex differences in SI among TMDs patients. Park additionally found that perceived stress and SI were both correlated in women with TMDs. Although these studies have offered initial insights into the factors influencing SI, the pathways remain unexplored. Therefore, mediation models could provide a clearer understanding of the relationship between chronic pain, psychological factors, and SI, thereby aiding in the identification of potential intervention targets.

This study aims to explore the factors associated with SI in TMDs, the role of TMDs related quality of life (TMD-QoL) in anxiety and SI, and the relationship between the three. Specifically, the hypotheses of this study are as follows: (1) Anxiety can influence TMD-QoL. (2) Anxiety and TMD-QoL may jointly contribute to SI. (3) TMD-QoL mediates the relationship between anxiety and SI.

## Methods

### Study design

This cross-sectional study was conducted at West China Hospital of Stomatology, from September 1, 2021 to December 16, 2024. Ethical approval was obtained from the Ethics Committee of West China Hospital of Stomatology (No. WCHSIRB-CT-2021-431), and all procedures were carried out in accordance with the Declaration of Helsinki. Before participation, all subjects provided written informed consent after receiving a full explanation of the study objectives and procedures.

### Participants

Patients with TMD-related complaints were consecutively recruited. The inclusion criteria for the study included (1) age above 12 years old; (2) diagnosis of TMDs; and (3) having cognitive and ability to complete the questionnaire independently. Exclusion criteria were (1) those who had a history of painkillers taken; and (2) a history of psychiatric disorders.

### Demographic information

Basic demographic information about the patients including age, sex, education level, and income level was collected for analysis. Educational level was categorized according to “below high school”, “college” and “postgraduate”. Income level was categorized according to the per capita monthly income of the household as “below 3,000 RMB”, “3,000–6,000 RMB” and “above 6,000 RMB”.

## Questionnaire collection

### Psychological assessment

The ninth item of the Patient Health Questionnaire − 9 Item (PHQ-9), which was developed by Kroenke, Spitzer, and Williams in 2001 [15], with excellent internal consistency (Cronbach’s α = 0.89) and test–retest reliability (kappa = 0.84 over 48 h), and criterion validity indicated by 88% sensitivity and 88% specificity against clinician interviews in the original validation (*n* ≈ 6,000) [16], “Thoughts that you would be better off dead or of hurting yourself in some way” was used to assess whether the patients had SI. Patients were considered suicidal if they chose the option “Several days”, “More than half the days”, or “Nearly every day” [17].

The severity of anxiety was assessed using the Generalized Anxiety Disorder Scale − 7 Item (GAD-7), which was created by Spitzer et al. in 2006, with excellent internal consistency (Cronbach’s α = 0.92) and test–retest reliability (intraclass correlation = 0.83), as well as strong procedural validity against clinician-administered scales [18]. The traditional Chinese version was validated among Taiwanese patients with epilepsy (*n* = 109), exhibiting high internal consistency (Cronbach’s α = 0.928) and robust construct validity [19, 20]. Patients rated how often they were bothered by symptoms in the past two weeks from 0 (not at all) to 4 (almost every day), with a total score threshold of 5, which was considered to be anxiety [21]. In our cohort, the GAD-7 yielded a Cronbach’s α of 0.931.

### Quality of life assessment

The Oral Health Impact Profile for Temporomandibular Disorders (OHIP-TMD) is a 22-item, condition-specific adaptation of the OHIP-49 originally developed by Slade and Spencer (1994) and refined by Durham et al. in 2011 to focus on TMD-related impacts across seven domains: functional limitations (2 items), physical pain (5 items), psychological discomfort (4 items), physical disability (2 items), psychological disability (5 items), social disability (2 items), and handicap (2 items) [22]. Scores for each item ranged from 0 (never) to 4 (very frequently). The putative OHIP-TMD reported Cronbach’s α = 0.94, confirming high internal consistency [22]. The Chinese version underwent forward–back translation and cultural adaptation, with construct validity supported by factor analysis and convergent validity evidenced by significant correlations with a global oral health rating; internal reliability was excellent (Cronbach’s α = 0.917) and test–retest (intraclass correlation = 0.899) [23]. The total TMD-OHIP score was divided into three intervals, and when the total score was higher than 66.67% of the total sample’s scores, which was higher than 22, the patient was considered to have had an impact on the TMD-QoL [24]. A domain was considered to be impacted if the average score for that was higher than 2 [25]. In this study, the OHIP-TMD showed a Cronbach’s α of 0.947.

### Pain assessment

The patient’s joint or masticatory muscle pain level was assessed using a numeric rating scale (NRS) with a score ranging from 0 (no pain) to 10 (worst possible pain). The original NRS demonstrated excellent test–retest reliability (intraclass correlation = 0.97) and construct validity through strong correlations with the Visual Analog Scale (*r* ≈ 0.94) in osteoarthritis patients [26]. Chinese-language administration yielded robust test–retest (intraclass correlation = 0.95) over 24 h [27]. According to the pain scale, a total score of 1–3 is considered mild pain, 4–6 is moderate pain, 7–9 is severe pain, and 10 is the worst possible pain. If the patient’s score was higher than 4, the patient was considered to be experiencing a more significant pain, or the pain had interfered with daily life [28, 29].

### Statistical analysis

Statistical analyses were conducted in Jamovi, version 2.6.19, *p* < 0.05 was considered statistically significant. Summary statistics of the demographic characteristics of the study sample were calculated as mean ± SD or count and percentage. For continuous variables, the Mann-Whitney U test was used for comparative analysis. For categorical variables, the chi-square test was used for analysis.

Multicollinearity among predictors was evaluated using Variance Inflation Factors (VIF). A conservative threshold of VIF < 5 was adopted to indicate acceptable collinearity. Spearman’s correlation was used to assess the relationship between each variable and SI. Subsequently, logistic regression was performed to examine the effect of each variable on SI. The logistic regression analysis was conducted in two models: an unadjusted model (Model 1) and an adjusted model (Model 2). In model 2, relevant demographic variables (e.g., age, income, gender, and education level) were included as covariates to control for potential confounding. Generalized linear models were used to assess the relationship of each variable with TMD-QoL and adjusted using the same covariates.

Mediation analysis was conducted with anxiety as the predictor, SI as the outcome, and pain and TMD-QoL as potential mediators, using the bias-corrected bootstrapping method (5, 000 resamples). If the 95% confidence interval (CI) of the path effect value does not include zero, it indicates that the effect is statistically significant.

## Results

**Fig. 1:**
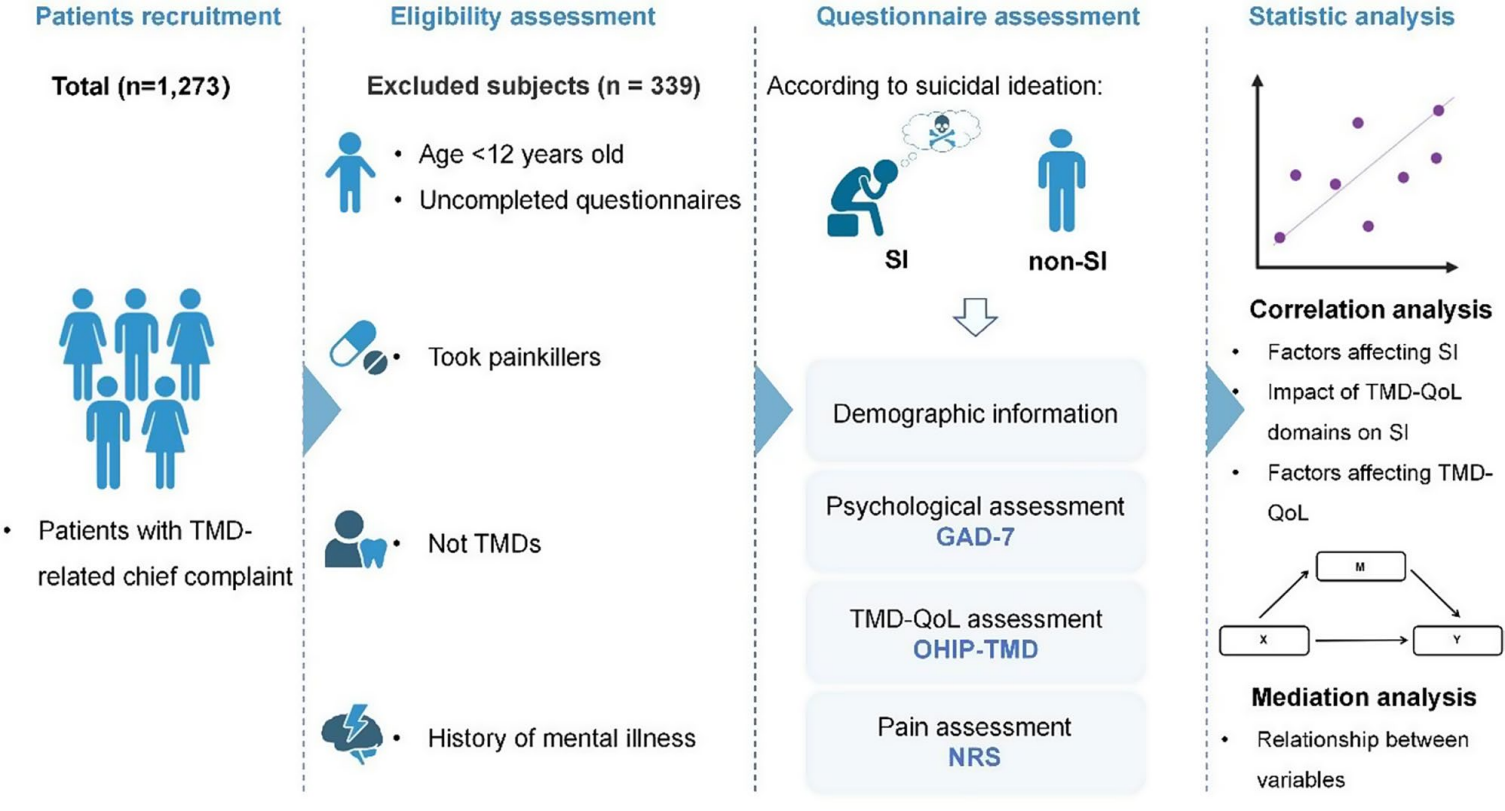
Flowchart for subject inclusion and information collectionAbbreviations: TMD, temporomandibular disorders; SI, suicidal ideation; GAD-7, generalized anxiety disorder-7 items; OHIP-TMD, oral health impact profile for temporomandibular disorders; NRS, numeric rating scal; TMD-QoL, temporomandibular-related quality of life

In this study, 934 patients with TMDs were included, with 69 patients in the SI group and 865 in the non-SI group (Fig. 1). The mean age of the SI group was 24.9 ± 8.9 years old, compared to 27.9 ± 11.3 years old in the non-SI group. Patients in the SI group were more likely to experience pain (NRS ≥ 4), suffer from anxiety (GAD-7 ≥ 5), and exhibit a greater impact on TMD-QoL (OHIP-TMD ≥ 29) compared to those in the non-SI group (Table 1).

**Table 1:** Characteristics of study participants according to SI

	non-SI	SI	Total
Participants, n	865	69	934
Age	27.9 ± 11.3	24.9 ± 8.9*	27.7 ± 11.1
Sex, male	187 (20.0)	13 (1.4)	200 (21.4)
Education			
≤ High school	237 (25.4)	23 (2.5)	260 (27.8)
College	528 (56.5)	40 (4.3)	568 (60.8)
≥ Postgraduate	100 (10.7)	6 (0.6)	106 (11.3)
Income			
≤ 3000 yuan	122 (13.1)	8 (0.9)	130 (13.9)
3000–6000 yuan	397 (42.5)	35 (3.7)	432 (46.3)
≥ 6000 yuan	346 (37.0)	26 (2.8)	372 (39.8)
Pain			
NRS<4	765 (88.44)	53 (76.81)*	818 (87.6)
NRS ≥ 4	100 (11.56)	16 (23.19)*	116 (12.4)
Anxiety			
GAD-7<5	575 (66.47)	14 (20.29)*	589 (63.1)
GAD-7 ≥ 5	290 (33.53)	55 (79.71)*	345 (36.9)
Impact on TMD-QoL			
OHIP-TMD<29	439 (50.75)	16 (23.19)*	455 (48.7)
OHIP-TMD ≥ 29	426 (49.25)	53 (76.81)*	479 (51.3)

Data are presented as mean ± SD or count (%). p-values were obtained by Mann-Whitney U test (continuous variables) and χ^2^ tests (categorical variables), and compared suicidal individuals with those who were not suicidal

Abbreviations: SI, suicidal ideation; NRS, numeric rating scale; GAD-7, generalized anxiety disorder-7 items; TMD-QoL, temporomandibular-related quality of life; OHIP-TMD, oral health impact profile for temporomandibular disorders

*Statistically significant difference (*p* < 0.05)

### Correlation between SI and variables

According to the results of Spearman’s correlation, age was negatively correlated with SI, while pain (NRS ≥ 4), impact on TMD-QoL (OHIP-TMD ≥ 29), and anxiety (GAD-7 ≥ 5) were positively correlated with SI (*p* < 0.05). Further logistic regression analysis demonstrated that anxiety was positively associated with the risk of SI (OR = 6.74, *p* < 0.001) (Table 2), which remained even after adjustment (OR = 6.70, *p* < 0.001) (Table 3).

**Table 2:** Spearman correlation and non-adjusted logistic regression with SI as dependent variable

	Correlation		Logistic regression		
	*r*	*p*	OR	95% CI	*p*
Age	−0.07	0.04*	0.96	0.93–0.99	0.01*
Sex	0.02	0.59			
Male			Reference		
Female			0.94	0.49–1.82	0.87
Education	-0.04	0.24			
≤ High school			Reference		
College			0.69	0.39–1.24	0.22
≥ Postgraduate			0.58	0.21–1.57	0.28
Income	-0.002	0.05			
≤ 3000 yuan			Reference		
3000–6000 yuan			1.87	0.81–4.29	0.14
≥ 6000 yuan			1.99	0.82–4.82	0.13
Pain	0.09	0.005**			
NRS<4			Reference		
NRS ≥ 4			1.51	0.79–2.88	0.22
Impact on TMD-QoL	0.14	< 0.001***			
OHIP-TMD<29			Reference		
OHIP-TMD ≥ 29			1.41	0.73–2.74	0.31
Anxiety	0.25	< 0.001***			
GAD-7<5			Reference		
GAD-7 ≥ 5			6.74	3.46–13.13	< 0.001***

Abbreviations: SI, suicidal ideation; NRS, numeric rating scale; GAD-7, generalized anxiety disorder-7 items; TMD-QoL, temporomandibular-related quality of life; OHIP-TMD, oral health impact profile for temporomandibular disorders; CI, confidence interval; r, correlation coefficient; β, regression coefficient; OR, odds ratio

*p* < 0.05*; *p* < 0.01**; *p* < 0.001***

**Table 3:** Adjusted logistic regression with SI as dependent variable

	OR	95% CI	*p*
Pain			
NRS<4	Reference		
NRS ≥ 4	1.61	0.84–3.10	0.15
Impact on TMD-QoL			
OHIP-TMD<29	Reference		
OHIP-TMD ≥ 29	1.60	0.82–3.14	0.17
Anxiety			
GAD-7<5	Reference		
GAD-7 ≥ 5	6.70	3.42–13.09	< 0.001***

Note: Including age, sex, education, and income as covariates

Abbreviations: SI, suicidal ideation; GAD-7, generalized anxiety disorder-7; NRS, numeric rating scale; TMD-QoL, temporomandibular-related quality of life; OHIP-TMD, oral health impact profile for temporomandibular disorders; CI, confidence interval; r, correlation coefficient; β, regression coefficient; OR, odds ratio; SI, suicidal ideation

*p* < 0.05*; *p* < 0.01**; *p* < 0.001***

### Correlation between SI and each domain of TMD-QoL

The results demonstrated that psychological discomfort, physical disability, psychological disability, social disability, and handicap were all positively associated with the risk of SI, with corresponding ORs of 3.45, 1.79, 3.61, 4.66, and 3.67, respectively (Table 4).

**Table 4:** Logistic regression of OHIP-TMD domain scores with SI as the dependent variable

	OR	95% CI	*p*
Functional limitation	1.61	0.97–2.69	0.065
Physical pain	1.58	0.89–2.78	0.112
Psychological discomfort	3.45	1.94–6.12	< 0.001***
Physical disability	1.79	1.09–2.92	0.021*
Psychological disability	3.61	2.19–5.96	< 0.001***
Social disability	4.66	2.67–8.13	< 0.001***
Handicap	3.67	2.22–6.05	< 0.001***

The OHIP-TMD assesses seven domains relevant to patients with TMDs. Domain was considered to be impacted if the average score for that was higher than 2. The relationship between SI and domain scores was assessed using binary logistic regression

Abbreviations: OHIP-TMD, oral health impact profile for temporomandibular disorders; SI, suicidal ideation; OR, odds ratio; CI, confidence interval; β, regression coefficient

*p* < 0.05*; *p* < 0.01**; *p* < 0.001***

### Correlation between TMD-QoL and variables

OHIP-TMD scores were used as the dependent variable to explore the factors affecting TMD-QoL, conducting a generalized linear regression model. The results showed that age, sex, pain, and anxiety were all positively associated with impact on TMD-QoL (all *p* < 0.05) (Table 5), and the association of pain and anxiety remained after adjustment (Table 6).

**Table 5:** Non-adjusted generalized linear model with “impact on TMD-QoL” as dependent variable

	β	95% CI	*p*
Age	0.16	0.08–2.40	< 0.001***
Sex			
Male	Reference		
Female	3.52	1.48–5.56	0.001**
Education			
≤ High school	Reference		
College	-2.09	-4.84-0.67	0.14
≥ Postgraduate	-2.01	-5.73-1.69	0.29
Income			
≤ 3000 yuan	Reference		
3000–6000 yuan	-0.27	-2.85-2.31	0.83
≥ 6000 yuan	-1.81	-4.51-0.90	0.19
Pain			
NRS<4	Reference		
NRS ≥ 4	9.99	7.51–12.49	< 0.001***
Anxiety			
GAD-7<5	Reference		
GAD-7 ≥ 5	16.10	14.39–17.81	< 0.001***

Abbreviations: TMD-QoL, temporomandibular-related quality of life; NRS, numeric rating scale; GAD-7, generalized anxiety disorder-7 items; CI, confidence interval; β, regression coefficient; OR, odds ratio

*p* < 0.05*; *p* < 0.01**; *p* < 0.001***

**Table 6:** Adjusted generalized linear model with “impact on TMD-QoL” as dependent variable

	β	95% CI	*p*
Pain			
NRS<4	Reference		
NRS ≥ 4	11.05	8.49–13.61	< 0.001***
Anxiety			
GAD-7<5	Reference		
GAD-7 ≥ 5	16.65	14.91–18.38	< 0.001***

Abbreviations: TMD-QoL, temporomandibular-related quality of life; NRS, numeric rating scale; GAD-7, generalized anxiety disorder-7 items; CI, confidence interval; β, regression coefficient; OR, odds ratio

*p* < 0.05*; *p* < 0.01**; *p* < 0.001***

### Mediation model

**Fig. 2:**
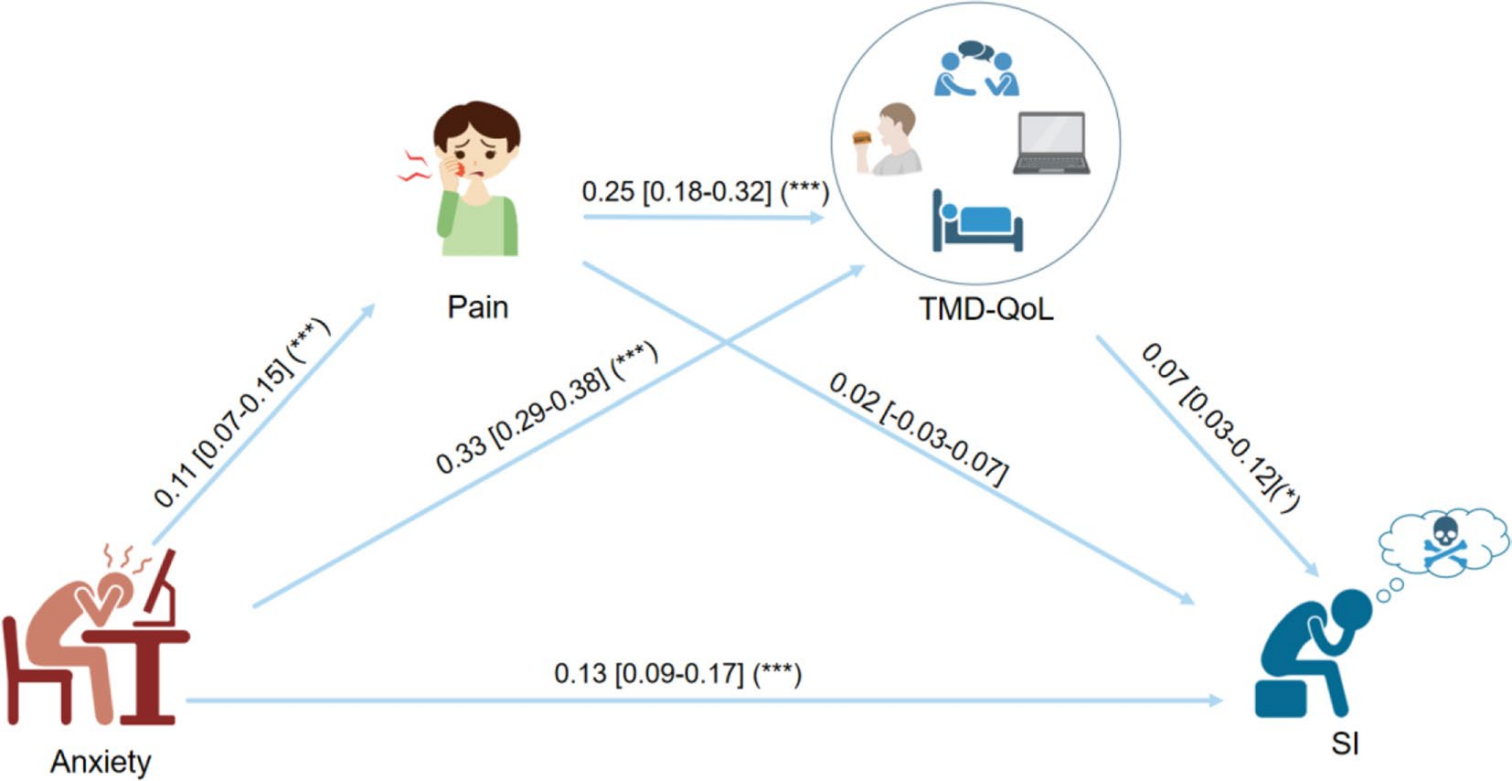
Mediation model of suicidal ideation Abbreviations: SI, suicidal ideation; TMD-QoL, temporomandibular-related quality of lifep < 0.05*; *p* < 0.01**; *p* < 0.001**

According to the mediation analysis, the effect of anxiety on SI was mediated through pain and TMD-QoL, with a total effect of 0.136 (Fig. 2). Anxiety had a positive effect on pain (0.11, 95%CI [0.07–0.15]), pain further influenced TMD-QoL (0.25, 95%CI [0.18–0.32]), and there was also a positive association between TMD-QoL and SI (0.07, 95%CI [0.03–0.12]). Meanwhile, anxiety could also affect SI through TMD-QoL (0.33, 95%CI [0.29–0.38]). In addition, anxiety also had a direct impact on SI (0.13, 95%CI [0.09–0.17]). These findings suggest that, in addition to directly affecting SI, anxiety could also exacerbate SI indirectly through its effects on pain and TMD-QoL (Table 7, Page 5).

**Table 7:** Effect coefficients of the mediator model

Variables	Pain			Impact on TMD-QoL			SI		
	β	95% CI	*p*	β	95% CI	*p*	β	95% CI	*p*
Anxiety	0.11	0.07–0.15	< 0.001***	0.33	0.29–0.38	< 0.001***	0.13	0.09–0.17	< 0.001***
Pain				0.25	0.18–0.32	< 0.001***	0.02	-0.03-0.07	0.44
Impact on TMD-QoL							0.07	0.03–0.12	0.002**

Note: The Mediator model has anxiety as the independent variable (X), suicidal ideation as the dependent variable (Y), and pain and impact on TMD-QoL as the mediator variable (M)

Abbreviations: TMD-QoL, temporomandibular-related quality of life; SI, suicidal ideation CI, confidence interval; β, regression coefficient; OR, odds ratio

*p* < 0.05*; *p* < 0.01**; *p* < 0.001***

## Discussion

This study explored the factors associated with SI and constructed a mediation model between them. Furthermore, the relationship between domains of TMD-QoL and SI and their potential influencing mechanisms were analyzed. The results showed that anxiety, pain, and TMD-QoL were all correlated with SI, while pain and TMD-QoL played a mediating role in the effect of anxiety on SI. In addition, this study also found that domains of TMD-QoL (including psychological discomfort, physical dysfunction, psychological dysfunction, social dysfunction, and handicap) were positively correlated with SI.

Regarding demographic characteristics, 21.4% of the participants were male, and 78.6% were female. This sex distribution aligns with the general trend of a higher prevalence of females among TMDs patients [30]. However, the distribution of SI did not differ significantly between sexes. The prevalence of SI in the total sample was 7.4%, and the mean age of the SI group was lower than that of the non-SI group (24.9 ± 8.9 years vs. 27.9 ± 11.3 years, *p* < 0.05), suggesting that younger individuals may be more susceptible to SI due to lower social adaptability or psychological resilience [31, 32]. Regarding clinical characteristics, the SI group exhibited higher proportions of both pain (NRS ≥ 4: 23.19% vs. 11.56%) and impact on TMD-QoL (OHIP-TMD ≥ 29, 76.81% vs. 49.25%) (*p* < 0.05), as pain is an independent risk factor for SI [33, 34]. This finding supports the potential role of multiple factors on SI in TMDs, which may affect mental health and TMD-QoL.

This study identified pain, TMD-QoL, and anxiety as key factors associated with SI in patients with TMDs. Further analysis revealed that individuals experiencing anxiety were more likely to exhibit SI, with an OR of 6.74. These findings align with previous studies, as a meta-analysis found that anxiety was associated with an increased risk of subsequent SI (OR = 1.97, 95% CI [1.72, 2.25]) [35]. And the severity of it could heighten the risk of suicidal behavior [36]. In the case of comorbidity, the odds of SI increased by 163% and the odds of suicidal behavior by 260% for each additional comorbidity [37]. Some studies suggested that this association may be since anxiety disrupts emotion regulation, making individuals more susceptible to external negative stimuli [38]. Specifically, anxiety may induce emotional instability by affecting the function of neurotransmitter systems, such as serotonin (5-HT) and gamma-aminobutyric acid [39, 40]. And the genetic variants related to anxiety, such as the 5-HT transporter gene and the brain-derived neurotrophic factor gene, may further heighten SI by influencing brain neuroplasticity and emotional regulation [41].

Furthermore, this study constructed a mediation model to reveal the possible regulatory pathways between anxiety and SI. The results showed that pain and TMD-QoL played a mediating role in this process. Specifically, anxiety was associated with SI through pain and TMD-QoL; it might also be related to SI only through TMD-QoL. The impact of psychological disorders on pain perception has been widely studied. Comparing the pain thresholds of patients with depression and anxiety, a study indicated that individuals with anxiety exhibited lower pain thresholds (effect size = -0.34) compared to those with depression (effect size = -0.60) [42]. And its severity is positively correlated with pain intensity [43]. TMDs are often accompanied by orofacial pain, and anxiety could influence pain sensitivity in patients [44, 45]. As studies indicate, this process could be associated with anxiety-induced activation of brain regions involved in pain perception [46]. Previous studies have shown that TMDs patients exhibit activation of the anterior cingulate cortex and insula, which are linked to pain perception, resulting in heightened pain intensity and persistence [47–49]. It may lead to an excessive focus on physical symptoms, which could impair daily functioning and reduce quality of life. On the other hand, anxiety may interfere with patients’ executive functioning, which could reduce flexibility in decision-making and problem-solving, consequently affecting quality of life [50, 51].

In addition, this study revealed the correlations between domains of TMD-QoL and SI, further elucidating the relationship between TMD-QoL and SI. Specifically, psychological discomfort, physical disability, psychological impairment, social limitation, and handicap were all significantly associated with SI, while psychological problems were more likely to be related to SI. Previous studies confirmed that psychological discomfort and social disability limit patients’ social interactions and support systems, and the lack of an effective social support may aggravate their psychological distress, leading to an inability to participate in normal daily activities, thus increasing the risk of loneliness and hopelessness, which is a risk factor for SI [52, 53]. Therefore, in addition to intervening in clinical symptoms and physical functioning of TMDs, it is also important to focus on patients’ psychological well-being.

This study also has several limitations that should be addressed in future research. First, despite the large sample size, the study was conducted at a single center, limiting the generalizability. Second, the cross-sectional design only captures data at a specific time point, preventing causal inferences between variables. Future research should employ longitudinal and multicenter designs to comprehensively understand the dynamic changes and interactions among relevant factors. Additionally, for clinical efficiency, this study utilized a brief psychological scale for assessment. However, such scales may have limitations in measurement accuracy and depth. Future research should incorporate more detailed and comprehensive psychological assessments to enhance the precision of psychological state evaluations. Moreover, this study used item 9 of the PHQ-9 to assess SI. While this method is commonly employed in clinical practice due to its simplicity and practicality, and previous studies have confirmed that it is an effective screening tool, it may underestimate the risk in individuals with more severe or long-term suicidal ideation [54, 55]. Future research should consider utilizing more comprehensive tools for assessment. In addition, the subjects of this study were all Chinese. Considering the differences between the cultures of countries, future studies need to further expand the sample types. Due to social stigma, patients may not report SI truthfully, thus reducing the probability of SI, future research should pay attention to communication with patients to reduce the impact. Finally, this study primarily focused on anxiety, whereas TMDs often present with a range of comorbidities, including sleep and somatic disorders. These factors may be closely linked to the onset and progression of TMDs. Future research should integrate these factors into analyses to develop a more thorough understanding of the pathological mechanisms and clinical characteristics of TMDs.

## Conclusion

This study identified anxiety, pain, and the impact of TMD-QoL as factors positively associated with SI in Chinese TMDs patients. Multiple dimensions of TMD-QoL were found to be related to SI. Furthermore, the relationship between anxiety and SI was mediated through a chain mediation model. These findings underscore the importance of incorporating psychological assessment into the clinical management of TMDs. Early identification of SI risk among TMDs may facilitate the development of targeted interventions. Future research should employ longitudinal cohort designs to investigate the factors influencing SI in TMDs and to elucidate the underlying mechanisms.

## Data Availability

The datasets used and/or analysed during the current study are available from the corresponding author on reasonable request.
